# Development of an observational - perceptual heat strain risk assessment (OPHSRA) index and its validation

**DOI:** 10.1186/s12889-021-12325-z

**Published:** 2021-12-30

**Authors:** Saeid Yazdanirad, Abbas Rahimi Foroushani, Mohammad Reza Monazzam, Habibollah Dehghan, Farideh Golbabaei

**Affiliations:** 1 School of Health, Shahrekord University of Medical Sciences, Shahrekord, Iran; 2 Modeling in Health Research Center, Shahrekord University of Medical Sciences, Shahrekord, Iran; 3 Department of Epidemiology and Biostatistics, School of Public Health, Tehran University of Medical Sciences, Tehran, Iran; 4 Department of Occupational Health Engineering, School of Public Health, Tehran University of Medical Sciences, Tehran, Iran; 5 Department of Occupational Health Engineering, School of Public Health, Isfahan University of Medical Sciences, Isfahan, Iran

**Keywords:** Heat stress, Risk assessment, Observational-perceptual index, Questionnaire

## Abstract

**Background:**

The thermal strain can be measured using subjective methods without the use of sensitive equipment. The purpose of the present study was the development and validation of an observational - perceptual heat strain risk assessment (OPHSRA) method.

**Methods:**

This cross-sectional study, in 2019, was performed. At first, an observational-perceptual questionnaire was designed using effective items in producing heat strain. Then, the reliability and validity of the questionnaire were examined. Later, 201 male workers were asked to perform the routine tasks for 90 min under various climatic conditions after resting in a cool room. At the end of the activity, the tympanic temperature of the subjects was accurately measured. Also, the designed questionnaire was completed by researchers and participants. Then, the effect coefficients of the items were calculated and used for developing the novel index. At final, the index validity was investigated.

**Results:**

The values of the content validity ratio (CVR), content validity index (CVI), and Cronbach’s coefficient alpha (α) of the designed questionnaire with 16 questions were equal to 0.793, 0.913, and 0.910, respectively. The results indicated that environmental, job, administrative, and clothing items assessed by the questionnaire with the coefficients of 0.860, 0.658, 0.783, and 0.566 had significant effects on the thermal strain, respectively. These coefficients were exploited to develop the index. The result revealed that the OPHSRA index justified 69% of the variations of the tympanic temperature (*R*^2^ = 0.69).

**Conclusion:**

The novel index developed by the questionnaire had an acceptable validity. Therefore, this index can be used for estimating the risk of thermal strain in a variety of thermal conditions.

## Background

Heat is one of the very common physical harmful agents in a variety of public and occupational environments, such as cement, steel, casting, and food produce industries. Heat exposure becomes a threat to people’s health [1]. Prolonged exposure to excessive heat stress can be led to an uncontrollable elevation in physiological responses, such as body temperature and heart rate, and increased risk of disorders and illness [2]. Some of these disorders included heat cramps, physical exhaustion, heat syncope, heatstroke, and even death [3]. Moreover, heat exposure can negatively affect the psychological, safety, socio-economics, and productivity aspects [4, 5]. Global warning enhances the thermal effects on people occupied in warm workplaces, particularly in hot climatic zones [6].

The efforts to quantify the risk of heat-related health effects have resulted in the development of more than one hundred heat stress indices [7]. The results of a review study performed by Freitas and Grigorieva revealed that there are 165 indices for evaluating heat strain imposed on bodies [8]. These indices can be generally divided into four groups, including environmental, physiological, perceptual, and perceptual-observational indices. Some environmental indices, such as wet bulb globe temperature (WBGT) and predicted heat strain (PHS), are extensively used around the world to predict the risk of heat strain [7]. However, these indices have several limitations. Those don’t consider individual differences such as age, gender, body mass, and acclimatization. Each of these indices is valid in the specific conditions of the studied parameters. Moreover, measuring them requires expensive equipment and skills [9]. Physiological indices such as physiological strain index (PSI) are another of the most common and valid methods to assess heat strain in the world [10]. However, measuring these indices requires direct contact with human and sensitive equipment. Accurate measurements in some sites such as rectal and esophageal are associated with stigma or invasiveness and high expense of some technologies [11]. Alternatively, subjective measures such as the thermal sensation scale and perceptual strain index (PeSI) were proposed to overcome these limitations [12]. The use of perceptual indices is increasing because of fast response, easiness, inexpensiveness, none-interference, and user-friendliness [13]. However, each of these indices takes into account one or a few human sensations on heat strain. Furthermore, individuals may make a mistake or exaggerate in expressing their perceptions. For resolving this issue, observational–perceptual methods were developed, which estimate the risk of heat strain through worker perception and expert assessment. However, this type of tool has received little attention, and a few numbers of them have been developed so far. Some of these known instruments include the checklist of scoring scales for observational assessment, observational checklist for heat stress risk assessment the checklist of basic thermal risk assessment, method of basic thermal risk assessment, method of work safety evaluation of hot and humid environments, and questionnaire of heat stress score index (HSSI) [14–18]. Table 1 describes the characteristics of some perceptual-observational instruments. Their common limitations include lack of assessing some important items (e.g. clothing thickness, covered body surface area, body movements, heat control measures, heat adaptation planning, and work-rest cycle), not having a scoring system, lack of categorizing the risk levels, and lack of evaluating the validity and reliability [18]. Therefore, a comprehensive tool for observational – perceptual risk assessment is required which does not possess these limitations. The purpose of the present study was the development and validation of an observational - perceptual heat strain risk assessment (OPHSRA) method.

**Table 1:** The characteristics of some perceptual-observational instruments

Instrument	Authors	Type	Year	Target population	Items	Risk levels	Validity and reliability
Scoring scales for observational assessment	Malchaire et al.	Observational and perceptual tool	1999	Male and female workers	Air temperature, humidity, thermal radiation, air movements, workload, clothing, and opinion of the worker	Without risk level	Those have been not evaluated.
Observational checklist for heat stress risk assessment	Bethea and Parsons	Observational and perceptual tool	2002	Male and female workers	Air temperature, radiant temperature, humidity, air velocity, metabolic rate, and clothing.	Without risk level	Those have been not evaluated.
Work safety evaluation of hot and humid environments	Zheng et al.	Observational and perceptual tool	2011	Male and female workers	Work nature, work intensity, work duration, temperature, humidity, airflow velocity, heat radiation intensity, seniority structure, safety training, and personal protection.	Four risk levels	Those have been not evaluated.
Instrument	Authors	Type	Year	target population	Items	Risk levels	Validity and reliability
Basic thermal risk assessment	Corleto et al.	Observational, perceptual, and measuring tool	2013	Male and female workers	Sun exposure, hot surface, exposure period, confined space, task complexity, climbing, up/down stairs or ladders, distance from cool rest area, distance from drinking water, permeability of clothing, understanding of heat strain risk, air movement, respiratory protection, acclimatization, metabolic work rate, and apparent temperature.	Three risk level	Those have been not evaluated.
Heat strain score index	Dehghan et al.	Observational and perceptual tool	2015	Male workers	Air temperature, surface temperature, air humidity, air movement, air condition, confined space, work location, physical activity, type of clothing, material of clothing, color of clothing, personal protective equipment, sweating rate, body posture, fatigue intensity, discomfort intensity, thirsty intensity, and clinical signs.	Three risk level	The content validity of this tool was confirmed. Cronbach’s coefficient (α) was calculated by 0.91.

## Methods

### Questionnaire development

#### Identification and categorization of effective items in producing heat strain

Effective items in producing heat strain were identified by a literature review through a search in known databases and interviews with the experts of occupational health. Then, those were reviewed and the repetitive and irrelative items were omitted. Improper items for designing the qualitative question were also eliminated. In final, 37 items remained in the study. Those were classified into six groups, including personal, environmental, job, administrative, clothing, and lifestyle items based on the balance theory of job design [19]. Based on this theory, a working system is made of five elements of individual, environment, task, tools, and technology, and organization. The balance between these five factors reduces the stress load. In the present study, personal items were considered as the individual factor, environmental items as the environment factor, job items as the task factor, clothing items as the tools and technology factor, administrative items as the organization factor. Additionally, lifestyle was added to these factors [20].

#### Questionnaire design

In this phase, a number of questions were generated for assessing the identified effective items. These questions were divided into three parts of observational, descriptive, and perceptual questions. Moreover, several responses were designed for each question. For quantifying the items, equivalent scores for each response were determined using subject-matter expertise, and later, those were modified based on the opinions of several experts. The draft questionnaire of the observational-perceptual heat strain risk assessment (OPHSRA) index included these questions. To develop the questionnaire, it was administered by the researchers. After developing the questionnaire, it can be administered by other people. The descriptive and perceptual questions are also answered by the workers.

#### Content validity evaluation

For evaluating the content validity, the questions were reviewed by ten experts with a research history on heat stress, including two professors, two associate professors, five assistant professors, and one Ph.D. candidate. They received an electronic mail, including the aims of the study and the draft questionnaire. The reviewers assessed the questions in terms of necessity using a 3-point Likert scale and in terms of relevance, clarify, and simplicity using the 4-point Likert scale. Lawshe and Waltz and Basel methods were applied to estimate the content validity ratio (CVR) and content validity index (CVI), respectively. The values of CVR and CVI greater than 0.79 and 0.62 were accepted, respectively [21, 22]. The questions with CVI values of 0.70 to 0.79 were also revised. Furthermore, averaged CVI and CVR of remained questions were calculated.

#### Reliability evaluation

For evaluating the reliability, the revised draft questionnaire was completed by 200 staff occupied in warm and dry and warm and humid areas, detailed below. Observational questions were also filled out by researchers, as experts. After, coefficients of Cronbach’s alpha (α) and McDonald’s omega were calculated for all questions and each group of questions. Moreover, the item-total correlation (ITC) coefficient of each question was computed, and the questions with ITC less than 0.3 were omitted. The ITC refers to the correlation between the item and the total scale. The minimum acceptable value of α was equal to 0.70 [23].

#### Back-translation evaluation

After preparing the final questionnaire in the English language, a back-translation was performed. For this purpose, a blind translator was asked to translate the questionnaire back into the Persian language. Then, an expert panel, including two English language specialists, two Persian language specialists, and three occupational health specialists with a research history on heat stress, compared the original and translated versions of the questionnaire and examined any discrepancies. If applicable, the panel redrafted the questions and answers until their concept, meaning, and quality became the same.

### Participants

Two hundred Iranian male staff (110 persons from a steel industry in the center of Iran as a hot and dry environment, and 90 persons from a petrochemical industry in the south of Iran as a hot and humidity ambiance) participated in the present study. It was tried that the subjects are selected from different industrial parts with a variety of climatic occupational conditions. For this purpose, the researchers attentively inspected the parts of these industries and elected the duties desired for performing the study. Then, the medical records of individuals working in these duties were investigated and the subjects with inclusion criteria were entered into the study. In the steel industry, these parts included forging, spark, induction melting, steelmaking, isolation, machining, refractory, technical support, engineering post, preventive maintenance, foundry, sandblast, metal waste separation, and administrative. In the petrochemical factory, the parts consisted of the warehouse, cookery, gardening, loading gantry, steel drum production, weighbridge, bitumen production, hydrocarbon, preventive maintenance, research and development, and administration. Inclusion criteria were career length higher than 1 year, no having mental, infectious, pulmonary, cardiovascular, hypertension, renal, hyperthyroidism, digestive, and diabetes diseases, non-use of medications to affect heart rate and blood pressure such as beta-blockers, phenothiazines, diuretics, anticholinergics, antispasmodics, psychotropics, antihistamines, antihypertensives, amphetamine, and decongestants, and non-use of coffee, caffeine, and alcohol from 12 h before the study. Furthermore, their tympanic membrane and auditory canal were medically screened. Exclusion criteria included unwillingness to impressive cooperation and body temperature higher than 39 °C during the activity.

### Sample size calculation

The aim of the present study was the development of an observational-perceptual index. The lowest correlation between the developed index and tympanic temperature was assumed as 0.2. Then, the sample size was computed based on the confidence level of 95% and a test power of 80% (Eq. 1).

1 $$n=\frac{{\left({Z}_{1-\frac{\alpha }{2}}+{\mathrm{Z}}_{1-\beta}\right)}^2}{w^2}+3\cong 194$$

Where ${Z}_{1-\frac{\alpha }{2}}$ is equal to 1.96 for a confidence level of 95%, *Z*_1 − *β*_ is equal to 0.84 for a test power of 80%, and W is equal to 0.203 for the lowest correlation coefficient of 0.2. Hence, the minimum sample size was obtained as 194 individuals.

### Study design and setting

In this cross-sectional study, data were gathered in the spring and summer seasons of 2019. Firstly, the subjects were asked to rest on a bed in a cool place around their workplace for 30 min. During this time, the steps of the study were explained to them and the demographical data of the subjects were collected. Also, their tympanic temperature was properly measured based on the standard of ISO 9886. After that, the participants were asked to return to their workplace and perform the routine tasks for 90 min. The researchers completed the observational questions during this time. At the end of 90 min, the tympanic temperature of the subjects was immediately and accurately measured based on the standard of ISO 9886. Simultaneously, they were asked to answer the descriptive and perceptual questions in the questionnaire. Moreover, environmental climatic parameters of dry temperature, wet temperature, globe temperature, relative humidity, and air velocity were recorded based on standards of ISO 7243 and ISO 7726. In the steel industry, the tasks performed by participants included refractory installation, metal waste separating, painting, welding, cutting, building, overhead crane operatory, overhead crane controlling, excavator driving, forklift driving, furnace operatory, casting operatory, pot operatory, sandblasting, molding, administrative activities, managing, monitoring, repairing, isolation operatory, and metalworking. In the petrochemical factory, the tasks performed by subjects consisted of cookery, cleaning, loading operatory, gardening, bitumen production operatory, hydrocarbon production operatory, drum carrying, welding, cutting, painting, pressing, administrative activities, managing, monitoring, forklift driving, repairing, building, and warehousing.

### Measurement instruments

The thermometer of Braun (IRT 6530 model with an accuracy of 0.1 °C) was used to measure the tympanic temperature. The WBGT meter (TES 1369B model with an accuracy of 0.1 °C) was applied for measuring the environmental climatic parameters of dry temperature, wet temperature, globe temperature, and relative humidity. Moreover, the developed questionnaire of OPHSRA was exploited to subjectively evaluate the effective items in producing heat strain through observation, description, and perception.

### Index development

The indirect effect coefficients of the items on thermal strain (variations of tympanic temperature) were calculated by structural equation modeling (SEM). Each of these coefficients was multiplied by the score of the related item, and resultant values were summed together for calculating the total score of the novel index.

### Data analyses

Gathered data were analyzed by the software of statistical package for the social sciences (SPSS) version 18. The normality of variables was examined using skew and kurtosis curves. Based on the results, all items had the normal distribution. Structural equation modeling (SEM) was applied to calculate the effect coefficients of the items. At first, the factor loadings of items of each factor, as direct effect coefficient, were computed using the Varimax method. Then, the regression method was used to calculate the score of each factor, in which factor loadings were multiplied by the data of each item. Later, a theoretical model was drawn using computed scores of the factors in AMOS software. The fitness of the designed model was evaluated using fit indices. Then, the novel index was developed by the indirect effect coefficients of the items in the model. Also, given the relationships between some of the items, it may be redundancy between them. In AMOS, modification Indices identified these redundant items. Co-variation was done between the measurement errors of redundant items based on the suggestion of the software for constraining the redundancy effects and increasing the fitness of the model. Finally, receiver operator curves (ROC) analysis was applied for categorizing the score of the novel index. Boundaries of risk levels included tympanic temperatures of 37.5, 38.0, and 38.5 °C [24]. In ROC curves, nearest points to the ideal state were considered as optimal cut-off points of the developed index. The validity of the index was also apprised using linear and quadratic regression analyses.

## Results

In total, 36 proper items affecting the heat strain were identified and classified into six groups. Those included personal items of skin color, body resistance, and effective diseases, environmental items of air temperature, air humidity, radiant temperature, thermal conduction, air velocity, wind direction, air pollution, and noise, job items of physical activity, mental workload, body movement, and body posture, administrative items of heat adaptation planning, heat exposure duration, work-rest cycle, shift work, work location (indoor or outdoor), heat control measures, access to cooling facilities, and access to cool rest room, clothing items of material, size, weave, thickness, color, ventilation, type (underwear use and covered body surface area), and personal protective equipment, and lifestyles items of smoking, salt consumption, drinking water, sleep situation, and work experience in a warm environment. Then, a draft questionnaire was designed for assessing these items.

In examining content validity, six items were eliminated, and 14 questions were revised. Furthermore, 14 items were omitted after evaluating the reliability of the questionnaire. The list of removed items is available in Table 8 in appendix B. In total, five questions of environmental factor, two questions of job factor, five questions of administrative factor, and four questions of clothing factor reminded. All questions of personal and lifestyle factors were completely removed. Table 2 reports the values of CVR, CVI, and ITC of the remained questions. Averaged CVR and CVI were calculated by 0.793 and 0.913, respectively. The results showed that the coefficients of Cronbach’s alpha and McDonald’s omega related to the final questionnaire with 16 questions were equal to 0.91 and 0.92, respectively. The values of Cronbach’s alpha of environmental, job, administrative, and clothing items were estimated as 0.81, 0.71, 0.85, and 0.89, respectively. The values of McDonald’s omega of environmental, job, administrative, and clothing items were also computed by 0.83, 0.71, 0.86, and 0.90, respectively. Based on the results, the validity and reliability of the questionnaire were at acceptable levels. The final version of this questionnaire with responses and scores is available in appendix A.

**Table 2:** The values of CVR, CVI, and ITC of remained questions

Type	Code	Factor	Questions	CVR	CVI	ITC^a^
Observational questions (completed by expert)	Q1	Job	Which parts of the person’s body are able to move while working?	0.630	0.909	0.417
	Q2	Administrative	How efficient is heat control measures, such as air conditioning and insulation, in the person’s workplace?	1.000	0.932	0.907
	Q3	Clothing	What material are the person’s work clothes made of?	0.630	0.932	0.522
	Q4	Clothing	How thick are the person’s work clothes?	0.630	0.841	0.654
	Q5	Clothing	Which one of the following items defines the person’s work clothes?	0.820	0.909	0.578
	Q6	Clothing	Which of the following protective equipment is used by the person while working?	1.000	0.977	0.576
Descriptive questions (completed by worker)	Q7	Administrative	How many days have passed since you were not present in a warm environment for a period of more than 3 days?	0.630	0.841	0.485
	Q8	Administrative	How many hours on average are you exposed to heat on a workday?	1.000	0.977	0.753
	Q9	Administrative	How many minutes on average do you rest in a cool environment in every 2 h working in a warm environment?	1.000	0.955	0.696
	Q10	Administrative	In which of the following environments do you work mostly?	0.820	0.841	0.537
Perceptional questions (completed by worker)	Q11	Environment	How do you feel about the air temperature in your workplace?	0.820	0.932	0.893
	Q12	Environment	How do you feel about the air humidity in your workplace?	0.820	0.886	0.457
	Q13	Environment	How do you feel about the thermal radiation on your skin in your workplace?	0.630	0.886	0.733
	Q14	Environment	How do you feel about the temperature during hand or foot contact with the equipment in your workplace?	0.630	0.886	0.640
	Q15	Environment	How do you feel about the air movement in your workplace?	0.630	0.955	0.508
	Q16	Job	What is the intensity of your physical activity while working?	1.000	0.955	0.548

^a^ ITC: correlation between item and total scale

**Fig. 1:**
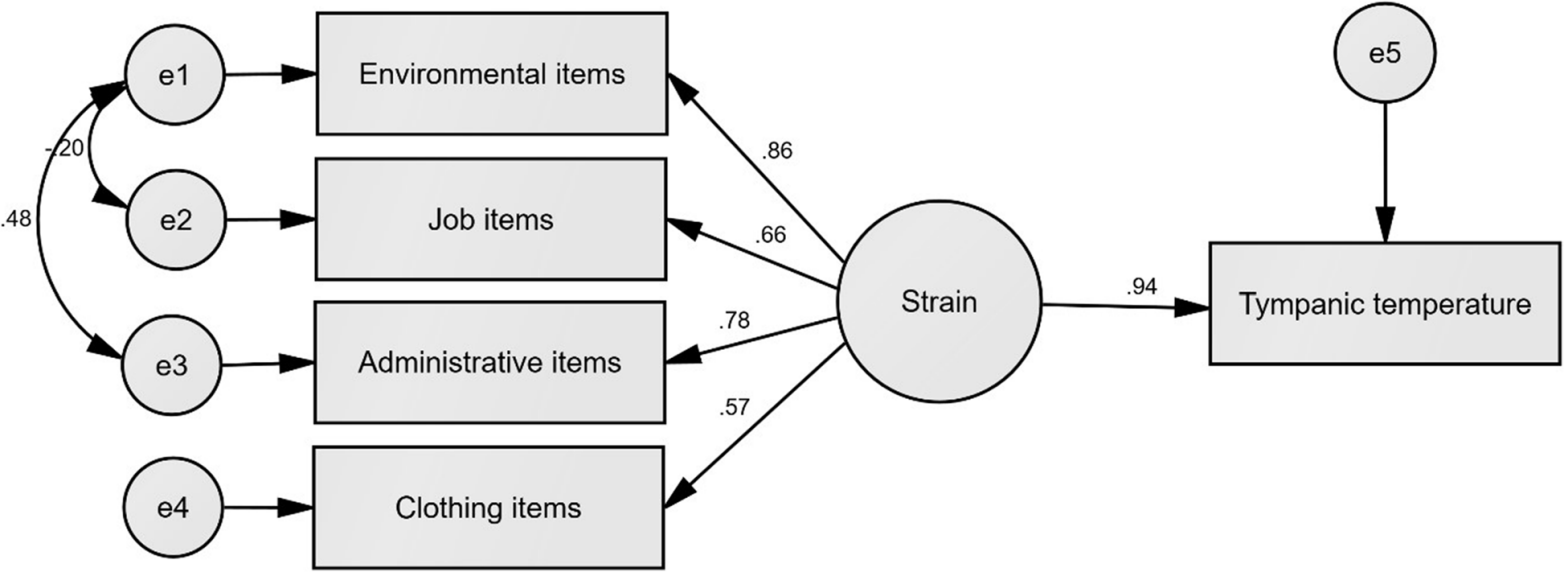
The theoretical model related to the impact of the factors in producing heat strain

This study results from a field survey involving 111 male employees of a steel factory (hot-dry ambiance) and 90 of a petrochemical factory (hot-humid environment). The values of the mean (standard deviation) of age, height, and weight were equal to 36.62 (8.24) years, and 1.76 (0.06) meters, and 80.52 (14.91) kilograms, respectively. Table 3 reports the statistical distribution of climatic parameters in the measured places. The results showed that each of the climatic variables encompassed a wide range of values. Table 4 also represents the statistical distribution of the items evaluated by the participants. Based on the results, the extensive ranges of scores related to the questions were collected for entering into the model. Figure 1 shows the theoretical model related to the impact of the items in producing heat strain. The authors assumed the structure in this figure. The results indicated that environmental, job, administrative, and clothing items with significant coefficients of 0.860, 0.658, 0.783, and 0.566 had significant effects on the thermal strain, respectively. Moreover, the results showed that one unit of increase in the thermal strain calculated by these factors enhance the mean tympanic temperature by 0.936. Table 5 describes the effect coefficients of the items. Of environmental items, the air temperature assessed by Q11 with a coefficient of 0.727 had the highest indirect effect on the tympanic temperature. Of job items, the body movement and physical activity assessed by Q1 and Q16 with a similar coefficient of 0.542 showed the highest indirect effect. Of administrative items, the greatest indirect effect belonged to the heat control measures assessed by Q2 with a coefficient of 0.673. Of clothing items, clothing thickness assessed by Q4 with a coefficient of 0.500 possessed the greatest indirect effect. Given that the administrative items such as heat control measures and job items such as physical activity can affect the perception of environmental parameters, there is redundancy between them. Modification indices in the software identified these redundant items. To constrain the redundancy effects and increase the model fitness, co-variation was done between the measurement errors of these items. Table 6 reports the goodness-of-fit indices of the analyzed model. Based on the results, the fitness of the presented model was confirmed.

**Table 3:** The statistical distribution of climatic parameters in the measured places

Parameter	Steel industry (*n* = 110)			Petrochemical industry (*n* = 90)		
	Range	Mean	Standard deviation	Range	Mean	Standard deviation
Dry temperature (degree of centigrade)	21.97–43.60	33.58	5.21	24.10–48.20	36.26	6.61
Wet temperature (degree of centigrade)	12.10–24.17	17.63	2.04	13.97–37.57	27.43	6.26
Globe temperature (degree of centigrade)	23.40–62.43	39.01	9.63	24.10–57.23	40.97	9.80
Relative humidity (percent)	9.01–39.31	19.60	9.92	14.82–79.11	52.09	17.71

**Table 4:** The statistical distribution of the evaluated items

Variable		Code	Range	Mean	Standard deviation
Environmental items	Air temperature	Q11	−1 – 4	2.33	1.32
	Relative humidity	Q12	0–4	1.34	1.33
	Radiant temperature	Q13	0–5	2.15	1.75
	Thermal conduction	Q14	-1 – 4	0.94	0.72
	Air velocity	Q15	0–3	1.09	0.42
Job items	Boby movement	Q1	0–4	1.71	0.88
	Physical activity	Q16	0–4	2.00	0.92
Administrative items	Heat control measures	Q2	0–4	2.62	1.39
	Heat adaptation planning	Q7	0–4	1.30	1.42
	Heat exposure duration	Q8	0–4	2.09	1.39
	Work–rest cycle	Q9	0–4	2.79	1.37
	Work location	Q10	1–3	1.66	0.92
Clothing items	Material	Q3	1–5	2.02	0.94
	Thickness	Q4	1–4	1.90	0.59
	Type	Q5	1–5	2.17	0.69
	Personal protective equipment	Q6	0–6	1.22	1.17

**Table 5:** The effect coefficients of the items

Variable		Code	Direct effect	Indirect effect	*P*-value
Environmental items	Air temperature	Q11	0.903	0.727	*P* < 0.001
	Relative humidity	Q12	0.606	0.488	
	Radiant temperature	Q13	0.836	0.673	
	Thermal conduction	Q14	0.799	0.643	
	Air velocity	Q15	0.685	0.551	
	Factor score	–	0.860	0.805	
Job items	Boby movement	Q1	0.880	0.542	*P* < 0.001
	Physical activity	Q16	0.880	0.542	
	Factor score	–	0.658	0.616	
Administrative items	Heat control measures	Q2	0.918	0.673	*P* < 0.001
	Heat adaptation planning	Q7	0.619	0.454	
	Heat exposure duration	Q8	0.901	0.660	
	Work–rest cycle	Q9	0.781	0.572	
	Work location	Q10	0.717	0.526	
	Factor score	–	0.783	0.733	
Clothing items	Material	Q3	0.906	0.480	*P* < 0.001
	Thickness	Q4	0.941	0.500	
	Type	Q5	0.887	0.470	
	Personal protective equipment	Q6	0.859	0.460	
	Factor score	–	0.566	0.530	
Thermal strain		–	0.936	–	*P* < 0.001

**Table 6:** Goodness-of-fit indices of the analyzed model

index	Name	Threshold of Fitness	Obtained value
Absolute fitness indices	Goodness-of-fit index (GFI)	> 0.9	0.991
	Adjusted goodness-of-fit index (AGFI)	> 0.9	0.955
Comparative fitness indices	Normed fit index (NFI)	> 0.9	0.993
	Comparative fit index (CFI)	> 0.9	0.998
	Incremental fit index (IFI)	0–1	0.998
Normed fit index	Root mean squared error of approximation (RMSEA)	< 0.1	0.049
	Normed Chi-square (X2/df)	1–3	1.483
*P* value		> 0.05	0.217

OPHSRA index was developed by the indirect effect coefficients of the items, as follow:

2 $${\displaystyle \begin{array}{c}\mathrm{OPHSRA}=\left[\left(0.542\times {\mathrm{Q}}_1\right)+\left(0.673\times {\mathrm{Q}}_2\right)+\left(0.480\times {\mathrm{Q}}_3\right)+\left(0.500\times {\mathrm{Q}}_4\right)\right]\\ {}+\left(0.470\times {\mathrm{Q}}_5\right)+\left(0.460\times {\mathrm{Q}}_6\right)+\left(0.454\times {\mathrm{Q}}_7\right)+\left(0.660\times {\mathrm{Q}}_8\right)\\ {}+\left(0.572\times {\mathrm{Q}}_9\right)+\left(0.526\times {\mathrm{Q}}_{10}\right)+\left(0.727\times {\mathrm{Q}}_{11}\right)+\left(0.488\times {\mathrm{Q}}_{12}\right)\\ {}+\left(0.673\times {\mathrm{Q}}_{13}\right)+\left(0.643\times {\mathrm{Q}}_{14}\right)+\left(0.551\times {\mathrm{Q}}_{15}\right)+\left(0.542\times {\mathrm{Q}}_{16}\right)\end{array}}$$

Where Q1 to Q16 are the scores of the questions in the final questionnaire (appendix A). It is important to note that the subjects can select several responses in questions of Q1 and Q6, and the sum of the scores of these answers is entered into the equation. Furthermore, the sign of the score of Q15 related to air movement changes from positive to negative when the score of Q11 related to the air temperature perception is lower than two because the heat strain decreases in these conditions.

**Fig. 2:**
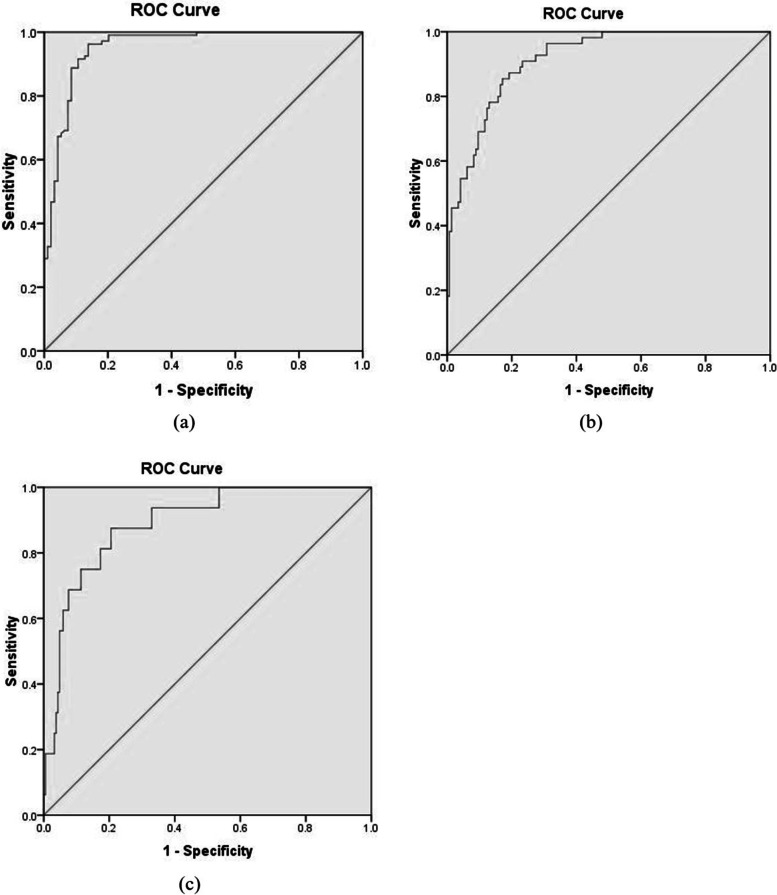
Receiver operating characteristic (ROC) curves related to **a** low and moderate risk zones, **b** moderate and high risk zones, and **c** high and very high risk zones

**Fig. 3:**
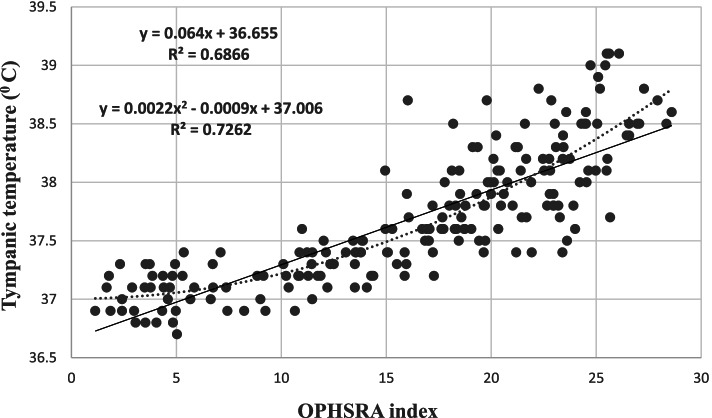
Linear and quadratic regression curves between tympanic temperature and OPHSRA index

Figure 2 indicates the curves of receiver operating characteristic (ROC) related to various risk zones. The results showed that optimal cut-off points of boundaries between low and moderate, between moderate and high, and between high and very high-risk zones were equal to 17.04 (sensitivity = 0.916 and specificity = 0.894), 20.06 (sensitivity = 0.829 and specificity = 0.885), and 22.10 (sensitivity = 0.875 and specificity = 0.795), respectively. Table 7 represents the risk levels and equivalent scores of the OPHSRA index. The area under of ROC curves (AUC) in Fig. 2a, b, and c were equal to 0.953 (95% CI: 0.923, 0.982) (*p* <  0.001), 0.915 (95% CI: 0.876, 0.953) (*p* <  0.001), and 0.890 (95% CI: 0.817, 0.963) (*p* < 0.001), respectively. Moreover, the validity of OPHSRA was investigated using the linear regression analysis between the developed index and tympanic temperature. Figure 3 displays the linear and quadratic regression curves between tympanic temperature and the OPHSRA index. The results of linear and quadratic regression analyses revealed that the OPHSRA index justified 69 and 73% of the variations of the tympanic temperature, respectively.

**Table 7:** The risk levels and equivalent scores of OPHSRA index

Risk level	Equivalent score
Low	Less than 17.04
Moderate	17.04 to 20.05
High	20.06 to 22.10
Very high	More than 22.10

## Discussion

In the present study, 37 effective items in producing thermal strain were identified and categorized into six groups, including personal, environmental, job, administrative, clothing, and lifestyle items. Zheng et al. identified ten items and classified them into three groups, including work, environment, and worker, for evaluating the safety under hot and humid conditions [16]. McLellan et al. also categorized the variables affecting the heat balance into four main groups, including the local environment, clothing, work intensity, and individual factors [25]. A higher number of effective items and factors in producing heat strain were identified in the present study compared to other studies. However, the designed questions related to 21 items were eliminated in the phases of examining validity and reliability. Finally, questions related to 16 items including air temperature, air humidity, radiant temperature, thermal conduction, air velocity, physical activity, body movement, heat adaptation planning, heat exposure duration, work-rest cycle, work location, heat control measures, clothing material, thickness, covered body surface area, and personal protective equipment reminded in the study. All questions of personal and lifestyle items were omitted. The values of CVR, CVI, Cronbach’s alpha, and McDonald’s omega of the observational – perceptual heat strain risk assessment (OPHSRA) questionnaire were calculated by 0.793, 0.913, 0.910, and 0.921, respectively. Based on the results, the validity and reliability of the questionnaire were at acceptable levels. In the heat stress score index (HSSI) questionnaire with 18 items designed by Dehghan et al., the value of Cronbach’s α was equal to 0.91. However, the values of CVI and CVR of this questionnaire were not reported [18].

This study was performed in the various climatic and occupational conditions so that the results revealed that the extensive ranges of scores related to observational, descriptive, and perceptual questions with normal distribution were collected. Therefore, this index can be applied for assessing the qualitative thermal strain risk of people occupied in different environments. In the model of the present study, environmental, administrative, job, and clothing items had significant effects on the thermal strain, respectively. Of these factors, the highest impact was related to the environmental items. In total, the main five items, including conductive heat, convective heat, radiant heat, sweat evaporation, and metabolism, impress on heat storage in the human body [26]. Therefore, the obtained finding is logical because the environment directly influences the perception of people on the first four items. Zheng et al. concluded that the working environment with a coefficient of 0.540 had the highest impact on the heat stress risk assessment process. Other factors included work and workers with the coefficients of 0.297 and 0.163, respectively [16]. For this reason, the main items were evaluated at many of the heat stress assessment indices such as Wet Bulb Global Temperature (WBGT) and Predicted Heat Stress (PHS). In the questionnaire of thermal environment and comfort assessment, questions have been mostly designed on environmental perceptions [27]. Moreover, the results of a study performed by Dehghan et al. showed that the highest correlations with the aural temperature were related to the environmental variables of the heat strain score index (HSSI) questionnaire [18]. These results are consistent with the results of the present study. Of environmental items, the perceptions of air temperature and humidity had the highest and lowest effect coefficients on the thermal strain, respectively. It may be because the other items, directly and indirectly, impress on the individual perception of air temperature. For example, the subjects have a warmer sensation of the air temperature during exposure to intense thermal radiation and high humidity. For this reason, known perceptual indices such as perceptual strain index (PeSI) applied the air temperature perception as a variable in assessing environment impact on subjective thermal strain [28]. While the results of the present study showed that the subjects were not too sensitive to the perception of humidity. Toftum et al. also resulted that the impact of humidity variation on the thermal comfort may be small in a certain range, and it becomes apparent under high-temperature conditions [29]. In the study of Dehghan et al., the variable of perceived air temperature showed the highest correlation with the aural temperature and the variables of perceived humidity level, and air movement had the lowest correlations among environmental items [18]. Of job items, questions related to body movement and physical activity had a similar effect coefficient because increased body movement can harmonically enhance the perceived physical activity. Of administrative items, the greatest indirect effect belonged to the question on the heat control measures. It is clear that heat control influences directly on climatic conditions and indirectly on body temperature. Other administrative solutions are not needed when heat control measures are completely implemented. Giahi et al. concluded that the control measures could effectively decrease the radiant heat of blast furnaces [30]. Of clothing items, thickness had the greatest indirect effect. It plays an important role in the thermal regulation of the body human. For this reason, the thickness and covered body surface area were used in the standard of ISO 9920 for estimating the thermal insulation of clothing assembles [31].

Based on the results, the fitness of the presented model was confirmed, and the diagnostic accuracies of ROC curves were at acceptable levels. Furthermore, the validity of the developed index was investigated using linear regression analysis. The results indicated that the OPHSRA index could justify 69% of the variations of tympanic temperature. In the study of Dehghan et al. [18], this value for the HSSI index with 18 items was equal to 51%. This study was performed on 122 male workers in two climatic conditions, including warm and dry and warm and humid environments in the industries. For evaluating the validity of this index, the subject carried out the routine tasks with different exercise intensity for 60 min after a rest period for 30 min. Then, they completed the questionnaire of HSSI and their aural temperature was measured. GOSS et al. also concluded that the OMNI scale of thermal sensation predicted 48% of variations in the core temperature. This study was conducted on 16 adult men and five adult women in a laboratory with air temperatures between 33 and 35 °C. The persons exercised on the treadmill with a speed of 4.5 km per hour for 50 min. In the end, they expressed their thermal sensation using an OMNI scale, and their core temperature was measured [32]. The results of a study performed by Dehghan and Ghanbari showed that PeSI justified 61% of the variations of oral temperature. This study was performed on 15 students in a climatic chamber with five different thermal stages of 21, 24, 27, 30, and 35 °C. In each stage, the individuals trained on a treadmill with low, moderate, and high physical activity intensity for 45 min, and then, they stated their thermal sensation and perceived exertion. Also, their oral temperature was measured [33]. These results demonstrate that the OPHSRA index possesses a higher validity compared to other objective indices for predicting the individual thermal strain. It may be because more items affecting thermal strain have been applied for developing the OPHSRA index. Moreover, the use of observational items reduces errors due to subjective perceptions. Also, the main items of heat strain in the workplace of each person can be identified using this index, and control measures can be focused on them. However, the novel index compared to objective environmental indices had a lower strength for predicting the tympanic temperature. Monazzam et al. concluded that WBGT and PHS indices justified 71 and 76% of the aural temperature, respectively. This study was conducted on 21 people working in the hot and humid sites of a petrochemical company. The parameters of WBGT and PHS indices were evaluated based on the standards of ISO 7243 and ISO 7933, respectively. Also, the body temperature of participants was measured [34]. This is logical because OPHSRA is calculated based on subjective judgment, while objective indices are computed using measurement device data. However, the OPHSRA index has a number of advantages, so that it can be exploited as a replacement tool for previous indices. This index, unlike objective indices such as WBGT, EHSRA [35], and PHSRA [36], can determine the risk of heat strain in the workers without the need for measurement devices. Also, it estimates the risk of heat strain before starting a job while some perceptual indices such as PeSI evaluate the risk during the activity. Moreover, the OPHSRA index comprehensively assesses several important risk factors for heat strain while some of them are not considered in previous indices. For example, the HSSI index doesn’t evaluate the risk factor of radiant temperature, clothing thickness, covered body surface area, body movements, heat control measures, heat adaptation planning, heat exposure duration, and work-rest cycle. Therefore, OPHSRA can be applied to reveal the main risk factors (items with high scores) in each of the workplaces so that the heat control measures are firstly focused on them. Additionally, the OPHSRA index can be used for screening people prone to thermal strain because individuals with different personal properties state various perceptions. Also, this index, unlike some observational–perceptual indices such as scoring scales for observational assessment and observational checklist for heat stress risk assessment, categorizes the risk and has an interpretation table for predicted heat strain level. Hence, the risk level can easily be determined after completing the OPHSRA questionnaire by the expert and worker and calculating the OPHSRA score using Eq. 2. However, each study has a few limitations. A limitation of the present study was the non-use of the OMNI scale and visual analog scale (VAS) to simplify the perceptual assessment. Moreover, it was not possible to exploit more accurate devices such as rectal thermometers because of ethical problems. Also, the sample was limited to males without predisposing medical conditions. Additionally, the validity of this questionnaire may be different in other cultures/languages.

## Conclusion

In total, the results of the present study showed that the designed observational perceptual questionnaire had acceptable validity and reliability. This questionnaire evaluates the environmental, job, administrative, and clothing items using 16 items. Based on the results, the novel index developed by this questionnaire showed an acceptable validity in the prediction of thermal strain. Therefore, this index can be used for estimating the risk of thermal strain and preventing the occurrence of heat-related illnesses in a variety of thermal conditions. However, it is suggested that validation of this index is investigated in other industries and the female workers. Its validity can also be studied in non-work environments.

## Data Availability

The datasets used and/or analyzed during the current study are available from the corresponding author on reasonable request.

## Appendix A

### Questionnaire of OPHSRA index

**Note:** The people can select several responses in questions of Q1 and Q6, and the sum of the scores of these answers is entered into the equation.

- **Observational questions (completed by expert)**

1. **Which parts of the person’s body are able to move while working?**
  - Head (1)
  - Trunk (1)
  - Hands (1)
  - Legs (1)
  - None (0)
2. **How efficient are heat control measures, such as air conditioning and insulation, in the person’s workplace?**
  - Very good (0)
  - Good (1)
  - Moderate (2)
  - Bad (3)
  - Very bad (4)
3. **What material are the person’s work clothes made of?**
  - One hundred percent natural fibers (1)
  - High percentage of natural fibers and low percentage of synthetic fibers (2)
  - High percentage of synthetic fibers and low percentage of natural fibers (3)
  - One hundred percent synthetic fibers (4)
  - Wool or fireproof fibers (5)
  - Impermeable material against steam, chemical, biological, and radionuclide substances (5)
4. **How thick are the person’s work clothes?**
  - Thin (1)
  - Moderate (2)
  - Thick (3)
  - Very thick (4)
5. **Which one of the following items defines the person’s work clothes?**
  - Regular clothes (non-use of work clothes) (1)
  - Short-sleeved work clothes (1.5)
  - Long-sleeved work clothes (2)
  - Long-sleeved work clothes with underwear (2.5)
  - Long-sleeved work clothes with coats or jackets (4)
  - Completely sealed work clothes with breathing apparatus (5)
6. **Which of the following protective equipment is used by the person while working?**
  - Safety helmet (1)
  - Earmuffs (0.5)
  - Non-cotton gloves (0.5)
  - Face shield (1)
  - Quarter face respirator mask (0.5)
  - Half face respirator mask (1)
  - Full face respirator mask (1.5)
  - Self-contained breathing apparatus (2)
  - Hood (1.5)
  - Boots (1)
  - Leather apron (1)
  - Aluminized clothing (− 1)
  - None (0)

- **Descriptive questions (completed by worker)**

7- **How many days have passed since you were not present in a warm environment for a period of more than three days?**
  - Less than four days (4)
  - Four to seven days (3)
  - Seven to ten days (2)
  - Ten to fourteen days (1)
  - More than fourteen days (0)
8- **How many hours on average are you exposed to heat on a workday?**
  - Less than two hours (0)
  - Two to four hours (1)
  - Four to six hours (2)
  - Six to eight hours (3)
  - More than eight hours (4)
9- **How many minutes on average do you rest in a cool environment for every two hours working in a warm environment?**
  - I don’t rest. (5)
  - Fifteen minutes (4)
  - Thirty minutes (3)
  - Forty-five minutes (2)
  - Sixty minutes and more (1)
  - I don’t work in a warm environment. (0)
10- **In which of the following environments do you work mostly?**
  - Indoors (1)
  - Outdoors (3)
  - Both environments (2)

- **Perceptual questions (completed by worker)**

11- **How do you feel about the air temperature in your workplace?**
  - Cool (− 1)
  - Temperate (0)
  - Slightly warm (1)
  - Warm (2)
  - Hot (3)
  - Very hot (4)
12- **How do you feel about the air humidity in your workplace?**
  - Dry (0)
  - Slightly humid (1)
  - Moderately humid (2)
  - Very humid (3)
  - Extremely humid (4)
13- **How do you feel about the thermal radiation on your skin in your workplace?**
  - Desirable (0)
  - Detectable but non-annoying (1)
  - Slightly annoying (2)
  - Moderately annoying (3)
  - Very annoying (4)
  - Burning and extremely annoying (5)
14- **How do you feel about the temperature during hand or foot contact with the equipment in your workplace?**
  - Cool (− 1)
  - Neutral (0)
  - Slightly warm (1)
  - Warm (2)
  - Hot (3)
  - Very hot (4)
15- **How do you feel about the air movement in your workplace?**
  - No air movement (0)
  - Light air movement (1)
  - Moderate air movement (2)
  - Strong air movement (3)
  - Very strong air movement (4)
16- **What is the intensity of your physical activity while working?**
  - Very light similar to resting (0)
  - Light (1)
  - Moderate (2)
  - Hard (3)
  - Very hard (4)

## Appendix B

**Table 8:** List of omitted items

Factor	Item	Reason of omission
Personal items	Skin color	CVR less than acceptable value
	Body resistance	ITC less than acceptable value
	Effective diseases	ITC less than acceptable value
Environmental items	Wind direction	CVR less than acceptable value
	Air pollution	CVR less than acceptable value
	Noise	CVR less than acceptable value
Job items	Mental workload	ITC less than acceptable value
	Body posture	ITC less than acceptable value
Administrative items	Shift work	CVR less than acceptable value
	Access to cooling facilities	ITC less than acceptable value
	Access to cool rest room	ITC less than acceptable value
Clothing items	Size	ITC less than acceptable value
	Weave	ITC less than acceptable value
	Color	ITC less than acceptable value
	Ventilation	ITC less than acceptable value
Lifestyles items	Smoking	CVR less than acceptable value
	Salt consumption	ITC less than acceptable value
	Drinking water	ITC less than acceptable value
	Sleep situation	ITC less than acceptable value
	Work experience in a warm environment	ITC less than acceptable value

## Abbreviations

WBGT: Wet bulb globe temperature
PHS: Predicted heat strain
PSI: Physiological strain index
PeSI: Perceptual strain index
HSSI: Heat stress score index
OPHSRA: Observational - perceptual heat strain risk assessment index
SEM: Structural equation modeling
CVR: Content validity ratio
CVI: Content validity index
α: Cronbach’s coefficient alpha
ITC: Item-total correlation
ROC: Receiver operator curves
VAS: Visual analog scale
